# Primary versus early secondary referral to a specialized neurotrauma center in patients with moderate/severe traumatic brain injury: a CENTER TBI study

**DOI:** 10.1186/s13049-021-00930-1

**Published:** 2021-08-04

**Authors:** Charlie Aletta Sewalt, Benjamin Yaël Gravesteijn, David Menon, Hester Floor Lingsma, Andrew I. R. Maas, Nino Stocchetti, Esmee Venema, Fiona E. Lecky, CENTER TBI Participants and Investigators, Cecilia Åkerlund, Krisztina Amrein, Nada Andelic, Lasse Andreassen, Audny Anke, Anna Antoni, Gérard Audibert, Philippe Azouvi, Maria Luisa Azzolini, Ronald Bartels, Pál Barzó, Romuald Beauvais, Ronny Beer, Bo-Michael Bellander, Antonio Belli, Habib Benali, Maurizio Berardino, Luigi Beretta, Morten Blaabjerg, Peter Bragge, Alexandra Brazinova, Vibeke Brinck, Joanne Brooker, Camilla Brorsson, Andras Buki, Monika Bullinger, Manuel Cabeleira, Alessio Caccioppola, Emiliana Calappi, Maria Rosa Calvi, Peter Cameron, Guillermo Carbayo Lozano, Marco Carbonara, Giorgio Chevallard, Arturo Chieregato, Giuseppe Citerio, Maryse Cnossen, Mark Coburn, Jonathan Coles, D. Jamie Cooper, Marta Correia, Amra Čović, Nicola Curry, Endre Czeiter, Marek Czosnyka, Claire Dahyot-Fizelier, Helen Dawes, Véronique De Keyser, Vincent Degos, Francesco Della Corte, Hugo den Boogert, Bart Depreitere, Đula Đilvesi, Abhishek Dixit, Emma Donoghue, Jens Dreier, Guy-Loup Dulière, Ari Ercole, Patrick Esser, Erzsébet Ezer, Martin Fabricius, Valery L. Feigin, Kelly Foks, Shirin Frisvold, Alex Furmanov, Pablo Gagliardo, Damien Galanaud, Dashiell Gantner, Guoyi Gao, Pradeep George, Alexandre Ghuysen, Lelde Giga, Ben Glocker, Jagoš Golubovic, Pedro A. Gomez, Johannes Gratz, Benjamin Gravesteijn, Francesca Grossi, Russell L. Gruen, Deepak Gupta, Juanita A. Haagsma, Iain Haitsma, Raimund Helbok, Eirik Helseth, Lindsay Horton, Jilske Huijben, Peter J. Hutchinson, Bram Jacobs, Stefan Jankowski, Mike Jarrett, Ji-yao Jiang, Kelly Jones, Mladen Karan, Angelos G. Kolias, Erwin Kompanje, Daniel Kondziella, Evgenios Koraropoulos, Lars-Owe Koskinen, Noémi Kovács, Alfonso Lagares, Linda Lanyon, Steven Laureys, Fiona Lecky, Rolf Lefering, Valerie Legrand, Aurelie Lejeune, Leon Levi, Roger Lightfoot, Hester Lingsma, Andrew I. R. Maas, Ana M. Castaño-León, Marc Maegele, Marek Majdan, Alex Manara, Geoffrey Manley, Costanza Martino, Hugues Maréchal, Julia Mattern, Catherine McMahon, Béla Melegh, David Menon, Tomas Menovsky, Davide Mulazzi, Visakh Muraleedharan, Lynnette Murray, Nandesh Nair, Ancuta Negru, David Nelson, Virginia Newcombe, Daan Nieboer, Quentin Noirhomme, József Nyirádi, Otesile Olubukola, Matej Oresic, Fabrizio Ortolano, Aarno Palotie, Paul M. Parizel, Jean-François Payen, Natascha Perera, Vincent Perlbarg, Paolo Persona, Wilco Peul, Anna Piippo-Karjalainen, Matti Pirinen, Horia Ples, Suzanne Polinder, Inigo Pomposo, Jussi P. Posti, Louis Puybasset, Andreea Radoi, Arminas Ragauskas, Rahul Raj, Malinka Rambadagalla, Ruben Real, Jonathan Rhodes, Sylvia Richardson, Sophie Richter, Samuli Ripatti, Saulius Rocka, Cecilie Roe, Olav Roise, Jonathan Rosand, Jeffrey V. Rosenfeld, Christina Rosenlund, Guy Rosenthal, Rolf Rossaint, Sandra Rossi, Daniel Rueckert, Martin Rusnák, Juan Sahuquillo, Oliver Sakowitz, Renan Sanchez-Porras, Janos Sandor, Nadine Schäfer, Silke Schmidt, Herbert Schoechl, Guus Schoonman, Rico Frederik Schou, Elisabeth Schwendenwein, Charlie Sewalt, Toril Skandsen, Peter Smielewski, Abayomi Sorinola, Emmanuel Stamatakis, Simon Stanworth, Ana Kowark, Robert Stevens, William Stewart, Ewout W. Steyerberg, Nino Stocchetti, Nina Sundström, Anneliese Synnot, Riikka Takala, Viktória Tamás, Tomas Tamosuitis, Mark Steven Taylor, Braden Te Ao, Olli Tenovuo, Alice Theadom, Matt Thomas, Dick Tibboel, Marjolein Timmers, Christos Tolias, Tony Trapani, Cristina Maria Tudora, Peter Vajkoczy, Shirley Vallance, Egils Valeinis, Zoltán Vámos, Gregory Van der Steen, Joukje van der Naalt, Jeroen T. J. M. van Dijck, Thomas A. van Essen, Wim Van Hecke, Caroline van Heugten, Dominique Van Praag, Thijs Vande Vyvere, Audrey Vanhaudenhuyse, Roel P. J. van Wijk, Alessia Vargiolu, Emmanuel Vega, Kimberley Velt, Jan Verheyden, Paul M. Vespa, Anne Vik, Rimantas Vilcinis, Victor Volovici, Nicole von Steinbüchel, Daphne Voormolen, Petar Vulekovic, Kevin K. W. Wang, Eveline Wiegers, Guy Williams, Lindsay Wilson, Stefan Winzeck, Stefan Wolf, Zhihui Yang, Peter Ylén, Alexander Younsi, Frederik A. Zeiler, Veronika Zelinkova, Agate Ziverte, Tommaso Zoerle

**Affiliations:** 1 Department of Public Health, Erasmus MC Medical Center, Postbus 2040, 3000 CA Rotterdam, The Netherlands; 2 Department of Anesthesiology, Erasmus MC Medical Center, Rotterdam, The Netherlands; 3 Division of Anaesthesia, Addenbrooke’s Hospital, University of Cambridge, Cambridge, UK; 4 Department of Neurosurgery, Antwerp University Hospital, and University of Antwerp, Edegem, Belgium; 5 Department of Pathophysiology and Transplantation, Milan University, and Neuroscience ICU, Fondazione IRCCS Cà Granda Ospedale Maggiore Policlinico, Milan, Italy; 6 Department of Neurology, Erasmus MC Medical Center, Rotterdam, The Netherlands; 7 Center for Urgent and Emergency Care Research (CURE), Health Services Research Section, School of Health and Related Research (ScHARR), University of Sheffield, Sheffield, UK

**Keywords:** Traumatic brain injury, Referral, Transfer, Trauma system

## Abstract

**Background:**

Prehospital care for patients with traumatic brain injury (TBI) varies with some emergency medical systems recommending direct transport of patients with moderate to severe TBI to hospitals with specialist neurotrauma care (SNCs). The aim of this study is to assess variation in levels of early secondary referral within European SNCs and to compare the outcomes of directly admitted and secondarily transferred patients.

**Methods:**

Patients with moderate and severe TBI (Glasgow Coma Scale < 13) from the prospective European CENTER-TBI study were included in this study. All participating hospitals were specialist neuroscience centers. First, adjusted between-country differences were analysed using random effects logistic regression where early secondary referral was the dependent variable, and a random intercept for country was included. Second, the adjusted effect of early secondary referral on survival to hospital discharge and functional outcome [6 months Glasgow Outcome Scale Extended (GOSE)] was estimated using logistic and ordinal mixed effects models, respectively.

**Results:**

A total of 1347 moderate/severe TBI patients from 53 SNCs in 18 European countries were included. Of these 1347 patients, 195 (14.5%) were admitted after early secondary referral. Secondarily referred moderate/severe TBI patients presented more often with a CT abnormality: mass lesion (52% vs. 34%), midline shift (54% vs. 36%) and acute subdural hematoma (77% vs. 65%). After adjusting for case-mix, there was a large European variation in early secondary referral, with a median OR of 1.69 between countries. Early secondary referral was not associated with functional outcome (adjusted OR 1.07, 95% CI 0.78–1.69), nor with survival at discharge (1.05, 0.58–1.90).

**Conclusions:**

Across Europe, substantial practice variation exists in the proportion of secondarily referred TBI patients at SNCs that is not explained by case mix. Within SNCs early secondary referral does not seem to impact functional outcome and survival after stabilisation in a non-specialised hospital. Future research should identify which patients with TBI truly benefit from direct transportation.

**Supplementary Information:**

The online version contains supplementary material available at 10.1186/s13049-021-00930-1.

## Background

Traumatic brain injury (TBI) remains an important cause of injury-related death and disability [1]. The incidence of TBI is increasing as the patient population becomes older [2, 3]. Care in specialized neurotrauma centers (SNC) with neurosurgical and neurocritical care expertise can reduce the incidence of death and disability from head injury, especially in more severe TBI [4–6]. However, not all TBI patients are directly transported to a SNC if this is not the nearest facility. In the prehospital setting Emergency Medical Services (EMS) should decide whether these patients should be stabilized at the nearby non specialist acute hospital (NSAH) or directly transported to a more distant SNC. After stabilization and computed tomography (CT) scan at a NSAH—the decision is made regarding the need for specialist neurotrauma care (including neurosurgery and neurointensive cares [7]) via secondary transfer. Stabilizing the patient at a nearby NSAH may cause an important time delay to critical neurosurgical and neurocritical care interventions which could adversely affect the outcome of TBI patients [8]. On the other hand prolonged primary transportation to a more distant specialist center could delay direct access to critical interventions such as drug assisted intubation, which prevents hypoxia and hypotension, that can induce secondary brain injury [9]. This is pertinent particularly to the majority of EMS staff who do not have this advanced airway skill [10]. Early neurosurgery might be a lower priority than early treatment of secondary insults such as hypoxia and hypotension [11]—the latter being addressed by hospital based damage control measures and balanced transfusion. The decision which patients should be conveyed directly to an SNC is made on-scene by EMS staff based on clinical parameters, injury characteristics and the local policy through trauma triage tools [10]. A systematic review on this issue failed to identify clear benefit from direct transportation to SNCs [11]. A recent randomized trial also failed to identify benefit as the majority of patients who bypass the NSAH are subsequently shown not to have a brain injury on CT scan, diluting the impact of early access to neurotrauma care [12, 13].

Notwithstanding this equivocal evidence base, several international guidelines recommend direct transportation of patients with moderate/severe TBI to hospitals with availability of neurosurgical care in order to reduce the time delay [14–16]. There might be substantial variation in referral practice between regions and countries. It remains unclear how long term outcomes of secondarily referred patients relate to outcomes of patients directly transported to a SNC, also in terms of secondary brain damage associated with hypoxia and hypotension.

Therefore, the aims of this study are, (1) to quantify European practice variation in early secondary referrals, and (2) to determine the association of arriving by early secondary referral with hypoxia and/or hypotension, survival at discharge and functional outcome at 6 months.

## Methods

### Study design

The Collaborative European NeuroTrauma Effectiveness Research in TBI (CENTER-TBI) study is a multicenter, longitudinal, prospective, observational study in 22 countries across Europe and Israel which enrolled patients between December 2014 and December 2017 [17]. All study sites are specialist neurotrauma centers [17]. The core cohort includes patients presenting within 24 h of injury, with a clinical diagnosis of TBI and indication for CT. Data for the CENTER-TBI study has been collected through the Quesgen e-CRF (Quesgen Systems Inc, USA), hosted on the INCF platform and extracted via the INCF Neurobot tool (INCF, Sweden).

We validated the generalizability of our analysis in the Center-TBI registry, comprising of all patients presenting at one of the study centers between December 2014 and December 2017 with a clinical diagnosis of TBI and indication for CT scan [17]. For the registry, informed consent was not necessary and collected purely administrative data which resulted in more included patients.

Version 2.1 of the core and registry Neurobot data sets were used for this study. Prehospital data was collected by physicians at the study centers. Policy and center specific data was collected by provider profiling questionnaires, filled in by the leading researchers of each study center [18]. Relevant questions from the provider profiling questionnaires to explain regional differences were the existence of a prehospital triage tool concerning direct transportation to more distant specialist neurotrauma centres and level of education of the prehospital staff.

Ethical approval was obtained for each recruiting site. Consent was obtained for all patients enrolled in the Core study. The list of sites, Ethical Committees, approval numbers and approval dates can be found on the website: https://www.center-tbi.eu/project/ethical-approval.

### Patient selection

We included all patients with moderate/severe TBI when presenting to the study center (defined as a Glasgow Coma Scale (GCS) < 13 or intubated [19]) who were transported by ambulance or helicopter directly to a study center (SNC) or admitted after early secondary referral within 24 h. Both patients with isolated TBI and polytrauma patients were included. A sensitivity analysis was done by including all CENTER-TBI registry patients with moderate/severe TBI. This study was reported in accordance with the STROBE reporting guidelines [20].

### Definitions

The outcome measures to estimate the effect of early secondary referral were hypoxia at ED arrival (saturation < 90%), hypotension at ED arrival (systolic blood pressure < 90 mmHg), survival at discharge and 6 months Glasgow Outcome Scale Extended (GOSE). For cases in which GOSE assessments had been performed outside the pre-specified window of 5–8 months, a multistate model was made by the CENTER-TBI statisticians to impute the 180-day GOSE [1, 21]. This imputed GOSE variable was made by the CENTER-TBI statisticians and was directly extracted from the CENTER-TBI Neurobot dataset [22]. This enables all CENTER-TBI researchers to use the same outcome variable. All other variables extracted from the CENTER-TBI Neurobot dataset were not imputed at the start of this research project. The following potential confounders between the relationship of transfer status and outcome were extracted from the CENTER-TBI Neurobot dataset because they were assumed to be associated with either arriving by early secondary referral or part of the International Mission for Prognosis and Analysis of Clinical Trials (IMPACT) model: age, GCS motor score at first Emergency Department (ED) arrival, pupil inequality at first ED arrival, hypoxia at ED arrival, hypotension at ED arrival, Injury Severity Score (ISS) and several CT abnormalities: traumatic subarachnoid haemorrhage (tSAH), epidural hematoma, mass lesion and acute subdural hematoma [23].

### Outcomes

Our primary outcome in order to quantify European practice variation is referral status (primary versus early secondary referral). Our secondary outcomes to determine the association between referral status and outcomes are 6 months GOSE, survival at discharge, hypoxia and hypotension. For the analysis in the CENTER-TBI registry, we used survival at discharge as outcome measure since longer term outcome data were not collected in the Registry.

### Statistical analysis

Continuous variables were described by the median and interquartile range (IQR). Categorical variables were described by the frequency and percentage. Missing data was imputed using multiple imputation, assuming missing at random (MAR). Missingness at random was assumed because the missingness present in our study can be accounted for by variables where there is complete information [24]. Missing data were multiply imputed for the main analyses using the ‘mice’ package. Together with the potential confounders mentioned above, referral status was included in the imputation model. Five imputed datasets were obtained. All variables, except for the outcome variables survival at discharge and the derived 6 months GOSE, were imputed. However, the outcome variables were included in the imputation model.

First, adjusted between-country differences were analyzed by adding a random intercept for country to a logistic regression model with early secondary referral as dependent variable. National variation or practice variation was quantified using the Median Odds Ratio [MOR, median odds ratio (OR) between two randomly picked countries/centers] [25].

Second, the effect of arriving by early secondary referral on hypotension and hypoxia was estimated using random effects logistic regression models. We adjusted for age, GCS motor score, pupil inequality, ISS and a random intercept for study center.

Third, the effect of arriving by early secondary referral on survival at discharge and functional outcome (6 months GOSE) was estimated using random effects regression models. For in-hospital mortality, a random effects logistic regression model was used, which included the predefined confounders and a random intercept for study center. For 6 months GOSE, a random effects ordinal regression model was used with similar structure. A subgroup analysis was done by including patients who presented with either a mass lesion or acute subdural hematoma on CT scan. This subgroup analysis used the same confounders to adjust for expect for CT abnormalities since this was the inclusion criterion of the subgroup analysis.

As a secondary sensitivity analysis in order to validate our results and assess generalizability, the same analysis was repeated in the CENTER-TBI registry with more heterogenous patients with survival at discharge as outcome measure. A random effects logistic regression model with the same case-mix variables was used with a random intercept for study center. Finally, as sensitivity analysis, the main analyses were also repeated in the complete cases only.

Continuous variables within the models were checked graphically for nonlinearity and were handled using restricted cubic splines when nonlinearity was assumed. Variables within models were checked for potential multicollinearity using correlation matrices. We did not check interactions terms because there were not enough degrees of freedom to do this.

Statistical analyses were performed in R statistical software 3.5.1 (R Foundation for Statistical Computation, Vienna). The *glmer* function from the *lme4* package was used for mixed effects logistic regression, the *clmm* function from the *ordinal* package was used for ordinal mixed effects logistic regression, and multiple imputation was performed using the *MICE* package.

## Results

### Patient characteristics

A total of 1347 patients with moderate/severe TBI were included in this study from 53 study centers in 18 European countries. Of these 1347 patients, 195 (14.5%) were transferred from another hospital. The proportion of TBI patients arriving through early secondary referrals varied by study center from 0 to 71%. The patients secondarily referred to the study center were mostly male (146, 74.9%), with a median age of 52 years (IQR 29–67), a median GCS of 7 (IQR 3–10), were not often intubated compared to primary referred in the prehospital environment (37, 20.7%) and their median ISS was 26 (25–41). The patients who were primarily transported to study centres were also mostly male (837, 72.7%), young to middle aged (median age was 47, IQR 28–65), with a GCS of 7 (4–10), however they were often intubated on-scene (701, 62.1%);their median ISS was 34 (25–45) (Table 1, Additional file 2: Figure S1). Mode of injury differed between both patient groups where road traffic incidents with extracranial injury were more common in TBI patients arriving by primary referral. When looking at the prehospital characteristics, patients secondarily referred had fewer on scene interventions (e.g. intubation, IV fluids) compared to primarily transported patients (Table 1).

**Table 1:** Patient characteristics, continuous: median (IQR), categorical: number (%); including percentage missingness for patient characteristics from core dataset (N = 1347)

	Primary referral (N = 1152)	% missing	Early secondary referral (N = 195)	% missing	*p* value
*Patient characteristics*					
Male (%)	837 (72.7)	0.0	146 (74.9)	0.0	0.52
Age (median [IQR])	47 [28, 65]	0.0	52 [29, 67]	0.0	0.34
Alcohol usage (%)	295 (29.0)	11.6	71 (42.3)	13.8	0.001
Drugs usage (%)	50 (5.4)	20.3	10 (7.4)	30.3	0.37
*Injury characteristics*					
Cause of injury (%)		10.9		15.9	< 0.001
Fall	420 (40.9)		84 (51.2)		
Road traffic incident	551 (53.7)		60 (36.6)		
Suicide	26 (2.5)		3 (1.8)		
Violence	30 (2.9)		17 (10.4)		
Area of injury = urban (%)	837 (75.0)	3.1	143 (76.5)	4.1	0.67
Place of injury (%)		2.3		5.1	< 0.001
Home	275 (24.4)		50 (27.0)		
Public location	66 (5.9)		35 (18.9)		
Sport	56 (5.0)		7 (3.8)		
Street	663 (58.9)		86 (46.5)		
Work	66 (5.9)		7 (3.8)		
GCS at arrival ED (median [IQR])	6 [3, 9]	0.0	7 [3, 10]	0.0	< 0.001
Hypoxia at arrival ED	186 (16.9)	0.0	18 (10.3)	0.0	< 0.001
Hypotension at arrival ED	196 (17.9)	0.0	20 (11.7)	0.0	< 0.001
Total ISS (median [IQR])	34 [25, 45]	0.7	26 [25, 41]	1.0	0.03
Major extracranial injury (AIS > 3) (%)	652 (57.0)	0.7	83 (43.0)	1.0	< 0.001
Pupil differences at ED (%)		3.4		6.7	0.10
No pupil difference	809 (72.7)		146 (80.2)		
One pupil not reactive	94 (8.4)		12 (6.6)		
Two pupils not reactive	210 (18.9)		24 (13.2)		
*Prehospital care*					
Intubation (%)	701 (62.1)	2.1	37 (20.7)	8.2	< 0.001
Ventilation (%)	646 (58.0)	3.4	35 (19.7)	8.7	< 0.001
CPR (%)	35 (3.0)	0.0	2 (1.0)	0.0	0.11
Oxygen supply (%)	853 (78.8)	6.1	97 (65.5)	24.1	< 0.001
IV fluids (%)	727 (63.1)	0.0	65 (33.3)	0.0	< 0.001
Physician on scene (%)	858 (74.7)	0.3	86 (44.6)	1.0	< 0.001
Mode of transport (%)		0.0		0.0	< 0.001
Ambulance	769 (66.8)		160 (82.1)		
Helicopter	271 (23.5)		16 (8.2)		
Medical mobile team	112 (9.7)		19 (9.7)		
Time from leaving scene to first hospital (median [IQR])	20 [12, 39]	41.8	11 [9, 24]	88	0.02
Time to study center (median [IQR])	20 [12, 39]	41.8	205[160, 286]	53	< 0.001
*Local policy characteristics*					
Prehospital triage protocol favours direct admission (%)	334 (46.7)	37.9	28 (27.2)	47.2	< 0.001
Training of prehospital staff (%)		19.4		24.1	0.61
BLS only	168 (18.1)		22 (14.9)		
Emergency medical technician	590 (63.6)		99 (66.9)		
Nurse	170 (18.3)		27 (18.2)		
*Imaging characteristics*					
Acute subdural hematoma (%)	700 (64.6)	5.9	131 (77.1)	12.8	0.004
Traumatic subarachnoid hemorrhage (%)	747 (73.6)	11.9	114 (76.5)	23.6	0.449
Epidural hematoma (%)	165 (16.2)	11.7	28 (18.7)	23.1	0.452
Skull fracture (%)	617 (59.9)	10.6	104 (65.8)	19.0	0.115
Midline shift (%)	377 (35.6)	8.0	91 (53.8)	13.3	0.007
Cisternal compression (%)	435 (41.6)	9.3	81 (50.6)	17.9	0.142
Mass lesion (%)	347 (34.1)	11.6	79 (52.0)	22.1	< 0.001
Intraventricular hemorrhage (%)	290 (28.6)	12.0	41 (27.0)	22.1	0.678
Contusion (%)	734 (69.3)	8.1	134 (79.3)	13.3	0.033
Emergency intracranial surgical intervention (%)	286 (24.9)	0.3	62 (32.1)	1.0	0.034
Time from injury to emergency surgery (median [IQR])	210 [150, 348]	37	345 [259, 479]	20.6	< 0.001
*Outcome*					
6 Months GOSE (median [IQR])	4.00 [1.00, 6.00]	12.7	4.00 [1.00, 7.00]	14	0.430
Unfavourable outcome (GOSE < 5, %)	491 (48.8)	12.7	62 (37.3)	14	< 0.001
In-hospital mortality (%)	190 (20.0)	17.7	30 (18.5)	16.9	0.610

Patients arriving after early secondary referral had more serious abnormalities on CT imaging; 131 (77.1%) of patients arriving by secondary transfer had an acute subdural hematoma, compared to 700 (64.6%) of the directly admitted patients; 79 (52.0%) of the referred patients had a mass lesion, compared to 347 (34.1%) of those arriving directly from the scene. The average time from injury to emergency surgery was approximately 210 min for directly admitted patients compared to 345 min when arriving by early secondary referral.

The median 6 months GOSE was 4 (IQR 1–6) among primary referred patients and 4 (IQR 1–7) among early secondary referred patients. In-hospital mortality was 21.2% among primary referred patients and 19.4% among secondarily referred patients.

### European practice variation of early secondary referrals

**Fig. 1:**
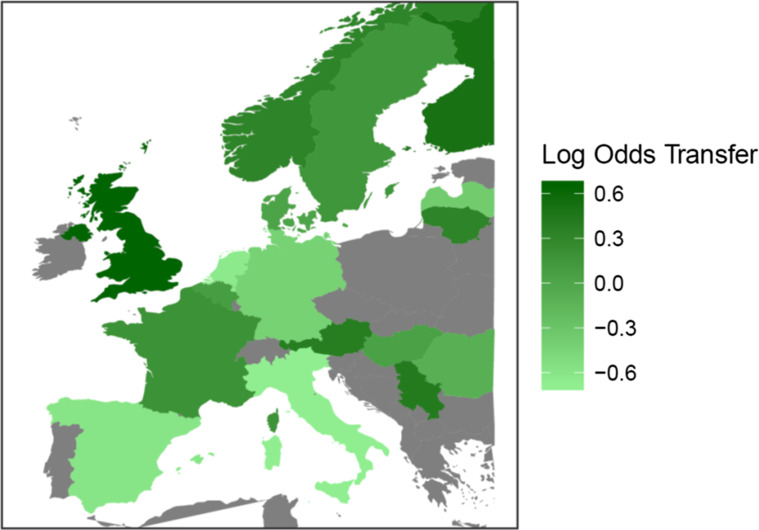
European practice variation in early secondary referrals, adjusted for extended IMPACT model (age, GCS motor score, pupil inequality, hypoxia, hypotension, ISS, CT lesions: tSAH, epidural hematoma, mass lesion, acute subdural hematoma). Log Odds represents the chance of arriving by early secondary referral for the mean moderate/severe TBI patient compared to the mean European chance of being referred. A log-odds above 0 means more chance than average of arriving by early secondary referral, a log odds below 0 means less chance than average of arriving by early secondary referral

When analysing European practice variation, patients admitted to specialist neurotrauma centers in Scandinavian countries, Austria and England were more often secondarily referred (Fig. 1). Patients in the Netherlands and Italy had relatively lower adjusted chance of arriving by early secondary referral. The MOR is 1.69 which means that the OR between two randomly picked countries is 1.69 for the average TBI patient included in our study.

### Effect of early secondary referral on outcome

There was no association between type of referral and hypotension and hypoxia at arrival at the SNC (unadjusted OR 0.53 with direct admission as reference, 95% CI 0.27–1.02 for hypoxia and OR 0.65 with direct admission as reference, 95% CI 0.36–1.19 for hypotension, Table 2). After adjustment, there were similar results (OR 0.57 with direct admission as reference, 95% CI 0.28–1.15 for hypoxia and OR 0.72 with direct admission as reference, 95% CI 0.38–1.38 for hypotension). Arriving by early secondary referral as moderate/severe TBI patient was not associated with 6 month GOSE (unadjusted OR 1.13 with direct admission as reference, 95% CI 0.82–1.55). After adjusting for confounders, arriving by early secondary referral as moderate/severe TBI patient was not associated with 6 month GOSE (multivariable adjustment, OR 1.07 with direct admission as reference, 95% CI 0.78–1.46, Table 3) and there was no association between early secondary referral and survival at discharge (OR 1.05 with direct admission as reference, 95% CI 0.58–1.90). Subgroup analysis of patients with a mass lesion or acute subdural hematoma and patients needing emergency intracranial surgical intervention showed similar magnitude and direction of the effects (Table 3). Complete case analysis showed similar results (Additional file 1: Table S3, Table S4).

**Table 2:** Effect of early secondary referral on hypotension and hypoxia at arrival at the Emergency Department of the Specialized Neurotrauma Center

	Hypoxia OR (95% CI)	Hypotension OR (95% CI)
Unadjusted	0.53 (0.27–1.02)	0.65 (0.36–1.19)
Multivariable adjustment^a^	0.57 (0.28–1.15)	0.72 (0.38–1.38)

There were 186 primary referred patients with hypoxia and 18 secondary referred patients with hypoxia. There were 196 primary referred patients with hypotension and 20 primary referred patients with hypotension

^a^Adjusted for: age, GCS motor score, pupil inequality, ISS and a random intercept for center

**Table 3:** Effect of early secondary referral on GOSE and survival at discharge

	6 months GOSE OR (95% CI)	Survival at discharge OR (95% CI)
Unadjusted	1.13 (0.82–1.55)	1.04 (0.65–1.62)
Multivariable adjustment^a^	1.07 (0.78–1.46)	1.05 (0.58–1.90)

Subgroup: patients with mass lesion/ASDH	N = 691	N = 651
Unadjusted	1.64 (1.10–2.44)	1.24 (0.67–2.32)
Multivariable adjustment^b^	1.28 (0.86–1.93)	1.02 (0.64–1.64)

Subgroup: patients with emergency intracranial surgical intervention	N = 301	N = 293
Unadjusted	1.51 (0.85–2.69)	1.59 (0.68–3.72)
Multivariable adjustment^b^	1.56 (0.89–2.74)	1.61 (0.56–4.76)

Higher OR for 6 months GOSE means better outcome, while higher OR for survival at discharge means higher chance of survival

^a^Adjusted for: age, GCS motor score, pupil inequality, hypoxia, hypotension, ISS, CT lesions: tSAH, epidural hematoma, mass lesion, acute subdural hematoma, and a random intercept for center

^b^Adjusted for: age, GCS motor score, pupil inequality, hypoxia, hypotension, ISS and a random intercept for center

### Sensitivity analysis in the registry

A total of 2150 moderate/severe TBI patients were included in the registry of which 25% arrived by secondary transfer, the characteristics of both groups were similar to patients in the core study (Additional file 1: Table S1). Secondarily referred patients had craniotomy for hematoma more often as emergency intervention [171 (10.5%) of directly admitted and 164 (31.4%) of secondarily referred patients]. Also, the CT scans of secondarily referred patients more frequently showed midline shift (54.5% for secondarily referred vs. 37.5% for directly admitted). There was no association between arriving by early secondary referral and survival at discharge after adjustment for confounders (OR 1.21 95% CI 0.84–1.73, Additional file 1: Table S2).

## Discussion

This study showed that variation in the proportion of moderate and severe TBI patients who have been secondarily referred to European specialist centres varied significantly by country after adjusting for case-mix factors. The secondarily referred TBI patients received less prehospital interventions. However, they had more serious abnormalities at CT scanning. Secondarily referred TBI patients were not associated with fewer secondary insults (hypoxia and hypotension at ED arrival). We found no association between early secondary referral and clinical long term outcomes. These findings were confirmed in the registry database, including a larger and more heterogenous population.

The European variation in the proportion of early secondary referrals to specialist centres is large, and only partly confirmed in previous literature. The likelihood of arriving by early secondary referral was lowest in the Netherlands and Italy. A previous Italian study showed that 58% of the TBI patients presenting at SNCs in the whole country were referred from another peripheral hospital [26]. However, Italian centers that contributed to CENTER-TBI were mainly situated in Northern Italy. Fifteen years ago, an English study found that one third of the severe head injury patients were treated in non-neurotrauma centers which was associated with higher mortality [27]. A study from Greece found that around half of the TBI patients in specialist centres were secondarily referred, higher than our findings. Early secondary referral increased the travel time to a neurosurgical center by 3.5 h [28]. The percentage of early secondary referrals seems to be decreasing when comparing our sample of moderate/severe TBI patients to older European studies. The percentage of secondarily referred patients was highest in Scandinavian countries, Austria and the UK. This is in line with their geography, less densely populated areas with long distances and the consequent need to stabilise their patients at closer non-specialised acute hospitals in order to avoid secondary insults.

Earlier research suggested that arriving by early secondary referral is associated with worse outcomes in severe TBI patients [8, 27, 29, 30]. One of the most important explanations for worse outcomes being the time delay which could result in secondary brain damage due to hypotension and hypoxia [31]. Also, care in centres that practice high-volume protocol-driven therapy, like ICP monitoring, is associated with better outcomes especially when neurocritical interventions are necessary [32, 33]. However, we could not find an effect of early secondary referral on long term outcomes. A meta-analysis including eleven studies found comparable results [12]. This is in line with previous research, suggesting that time interval to surgery was not associated with outcomes in patients with acute subdural hematomas requiring surgery [34]. Since subdural hematomas were the most prevalent CT abnormality in early secondary referred patients, these data suggest that these patients can safely be stabilised in non-specialised centers.

Our study shows that the impact of time to emergency surgery on outcomes becomes less critical when secondary insults (hypoxia and hypotension) are avoided. Hypoxia and hypotension are although less frequently observed over time in TBI patients still strongly associated with worse long term outcomes [35, 36]. We found no differences in secondarily referred TBI patients arriving with hypoxia or hypotension compared to directly admitted TBI patients at the Specialised Neurotrauma Centre. This is not in line with previous research which shows that interventions to treat life-threatening events may significantly decrease mortality [37]. We do see a non-significant association between arriving by secondary referral and less hypoxia or hypotension. We believe that shortened on-scene time and prompt transport to a non-specialist acute care facility where patients can be stabilized is associated with less hypoxia and hypotension when arriving at the specialized neurotrauma center. This is also in line with the trial of Bernard which shows that prompt intubation is associated with improved functional outcome in severe TBI patients [9]. When intubation is not possible in the prehospital field, it is important to transport the patient to the nearest acute care facility as soon as possible [35]. The reason for not finding a significant association might be the study power needed to show this association.

This study has several strengths. CENTER-TBI is a multicenter study in 22 European countries, which increases external validity. External validity is further increased because we were able to validate our findings for the effect of early secondary referral on outcome in the CENTER registry. We could rigorously adjust for potential case-mix differences due to the broad data collection of CENTER-TBI, and assess both survival and long term functional outcome.

However, our study also has several limitations. The biggest limitation of our study is the fact that we miss information about patients who died at the first hospital. This could introduce a selection bias where patients secondary referred to SNCs are the survivors of the first hospital they were admitted to. First, we could only include patients that were referred to a neurosurgical study center within 24 h after injury. Some moderate/severe TBI patients who may have benefited from specialised care might not have been transferred, or might have been transferred after 24 h. Late secondary transfers are associated with worse outcomes [38]. Second, inevitably our large multicentre prospective observational study meant data was missing for some variables and confounding bias could not be excluded. For example, time to first hospital was missing in 50% of the cases. This was addressed by using multiple imputation, a method proven to give valid estimates under the missing at random assumption [39]. We used random effects multivariable models to adjust for potential confounders and further adjust for potential between-center differences in study population. However, we cannot exclude residual confounding bias. Also, we adjusted for many factor which could also introduce over-adjustment. Potential confounding factors were selected on clinical reasoning and the IMPACT model. Third, the between-country and between-center differences could not be explained by the captured policy and care characteristics [18]. Fourth, since CENTER-TBI is a large multicentre prospective cohort study measurement errors were inevitable. We dealt with this by checking the dataset on impossible values (for example a heart rate of 999), and these values were checked in the patient records. When no mistake could be found, values were made missing. Fifth, regression dilution bias due to measurement error or random noise is possible. Last, geographical differences like the distance from scene to the specialised center, or the number of specialised neurosurgical centers per km^2^ were not measured at a patient level.

The debate about whether or not to transport TBI patients directly to specialist neurotrauma centers—past closer non specialist hospitals—has not yet been concluded. We were not able to find an association between early secondary referral and outcome. Intuitively, arriving by early secondary referral with extended time from injury to definitive treatment remains undesirable. One could look for alternatives. An English study shows for example that observation in a non-specialised hospital with neurosurgical consult by e-health and repeated CT scanning was not associated with worse outcomes for TBI patients [40]. However, this could lead to extra transfers between hospitals and increasing health care costs.

Once moderate/severe TBI patients are stabilised (on-scene or at the first hospital), it is possible that there is no effect of the time delay on outcome anymore. Patients arriving by early secondary referral receive less interventions on-scene, but do have more serious CT brain scan abnormalities, highlighting the limitations of current prehospital triage tools. Future research in this area also needs to include patients with TBI admitted to non-specialist hospitals. This will enable assessment of subgroups of TBI patients with benefit from direct transport to SNCs. Consequently, this would also allow further evaluation of the cost-effectiveness of direct transport to SNCs which was recently shown to be equivocal [41].

## Conclusions

Across Europe, substantial practice variation exists in the proportion of secondarily referred moderate/severe TBI patients within specialised neurotrauma centers. Patients who are secondarily referred present less often with secondary insults, although they have more serious CT abnormalities. Future research should focus upon which on scene characteristics identify TBI patients that benefit from direct transportation to distant specialist neurotrauma centers in order to improve guidelines and outcomes for patients with TBI.

## Supplementary Information

**Additional file 1.** Table S1 and S2.

**Additional file 2.** Figure S1.

## Data Availability

The data that support the findings of this study are available from https://www.center-tbi.eu but restrictions apply to the availability of these data, which were used under license for the current study, and so are not publicly available. Data are however available from the authors upon reasonable request and with permission of CENTER-TBI.

## Abbreviations

CI: Confidence interval
CT: Computed tomography
EMS: Emergency medical services
GCS: Glasgow Coma Score
GOSE: Glasgow Outcome Scale Extended
IQR: Interquartile range
NSAH: Non specialized acute hospital
OR: Odds ratio
SNC: Specialized neurotrauma center
TBI: Traumatic brain injury
