# Supplementary material for: Primary versus early secondary referral to a specialized neurotrauma center in patients with moderate/severe traumatic brain injury: a CENTER TBI study
Source: Scand J Trauma Resusc Emerg Med. 2021 Aug 4;29:113. doi: 10.1186/s13049-021-00930-1 (PMC8340517; doi:10.1186/s13049-021-00930-1)
Supplement: Supplementary file 1 — Additional file 1. Table S1 and S2. [file 13049_2021_930_MOESM1_ESM.docx]

**Appendices**

**Table S1:** Baseline characteristics for registry data including moderate/severe TBI patients only, continuous: median (IQR), categorical: number (%)

|  | Primary referral (N=1627) | Early secondary referral (N=523) | p-value |
| --- | --- | --- | --- |
| **Patient and injury characteristics** |  |  |  |
| Age (median [IQR]) | 52.00 [30.00, 71.00] | 57.00 [41.00, 73.00] | <0.001 |
| Male (%) | 1151 (70.7) | 374 (71.5) | 0.78 |
| Mechanism of Injury (%) |  |  | <0.01 |
| Assault | 120 (7.8) | 29 (6.2) |  |
| High energetic fall | 360 (23.5) | 120 (25.8) |  |
| Low energetic fall | 479 (31.2) | 187 (40.2) |  |
| Other | 8 (0.5) | 4 (0.9) |  |
| Road traffic incident | 535 (34.9) | 117 (25.2) |  |
| Self-harm | 32 (2.1) | 8 (1.7) |  |
| Place of injury (%) |  |  | <0.001 |
| Home | 556 (36.9) | 244 (50.7) |  |
| Public location | 231 (15.3) | 59 (12.3) |  |
| Sport | 27 (1.8) | 11 (2.3) |  |
| Street | 635 (42.1) | 150 (31.2) |  |
| Work related | 58 (3.8) | 17 (3.5) |  |
| GCS at arrival ED (median [IQR]) | 7 [3, 11] | 7 [3, 10] | 0.1 |
| SBP at arrival ED (median [IQR]) | 130 [113, 150] | 134 [119, 154] | <0.01 |
| Saturation at arrival ED (median [IQR]) | 99 [96, 100] | 98 [96, 100] | 0.24 |
| ISS (median [IQR]) | 25 [12, 41] | 25 [16, 33] | 0.20 |
| Pupil differences at ED (%) |  |  | 0.27 |
| No pupil difference | 1171 (75.5) | 385 (75.9) |  |
| One pupil not reactive | 128 (8.3) | 51 (10.1) |  |
| Two pupils not reactive | 251 (16.2) | 71 (14.0) |  |
| Intubation (%) | 977 (60.1) | 379 (72.5) | <0.001 |
| Mode of transportation (%) |  |  | <0.001 |
| Ambulance | 685 (42.9) | 187 (36.6) |  |
| Helicopter | 205 (12.8) | 18 (3.5) |  |
| Medical mobile team | 663 (41.5) | 275 (53.8) |  |
| None | 44 (2.8) | 31 (6.1) |  |
| **Emergency interventions** |  |  |  |
| Extraperitoneal pelvic packing (%) | 11 (0.7) | 2 (0.4) | 0.67 |
| Damage control laparotomy (%) | 24 (1.5) | 8 (1.5) | 1.000 |
| Damage control thoracotomy (%) | 17 (1.0) | 2 (0.4) | 0.25 |
| Decompression craniectomy (%) | 99 (6.1) | 50 (9.6) | <0.01 |
| Craniotomy for hematoma (%) | 171 (10.5) | 164 (31.4) | <0.001 |
| Interventional radiology (%) | 28 (1.7) | 4 (0.8) | 0.17 |
| External fixation limb (%) | 58 (3.6) | 12 (2.3) | 0.20 |
| **Imaging characteristics** |  |  |  |
| Intracranial lesions (%) | 984 (87.2) | 448 (90.3) | 0.083 |
| Subarachnoid hemorrhage (%) | 783 (69.8) | 322 (65.3) | 0.085 |
| Midline shift (%) | 405 (37.5) | 249 (54.5) | <0.001 |
| Intracranial contusion (%) | 666 (69.1) | 303 (69.2) | 1.0 |
| Subdural hematoma (%) | 665 (68.8) | 317 (70.9) | 0.47 |
| Epidural hematoma (%) | 171 (17.9) | 87 (19.8) | 0.45 |
| **Outcome characteristics** |  |  |  |
| In-hospital mortality (%) | 358 (23.5) | 101 (20.4) | 0.18 |

**Table S2:** Effect of early secondary referral on in-hospital mortality in the registry.

|  | Survival on discharge  OR (95% CI) |
| --- | --- |
| Unadjusted | 1.14 (0.87 – 1.49) |
| Multivariable adjustment* | 1.21 (0.84 – 1.73) |
| **Subgroup: patients with mass lesion/ASDH** |  |
| Unadjusted | 1.44 (1.05 – 1.97) |
| Multivariable adjustment** | 1.06 (0.70 – 1.58) |

*adjusted for: age, GCS, pupil inequality, ISS, CT lesions: tSAH, epidural hematoma, mass lesion, acute subdural hematoma, and a random intercept for center.

** adjusted for: age, GCS, pupil inequality, ISS and a random intercept for center.

**FigureS1: Strobe Flowchart**

**Table S3:** Complete case analysis. Effect of early secondary referral on hypotension and hypoxia at arrival at the Emergency Department of the Specialized Neurotrauma Center. There were 186 primary referred patients with hypoxia and 18 secondary referred patients with hypoxia. There were 196 primary referred patients with hypotension and 20 primary referred patients with hypotension.

|  | Hypoxia  OR (95% CI) | Hypotension  OR (95% CI) |
| --- | --- | --- |
| Unadjusted | 0.47 (0.25-0.85) | 0.62 (0.35-1.12) |
| Multivariable adjustment* | 0.50 (0.23-1.12) | 0.73 (0.34-1.58) |

*adjusted for: age, GCS motor score, pupil inequality, ISS and a random intercept for center.

**Table S4**: Complete case analysis. Effect of early secondary referral on GOSE and survival at discsharge. Higher OR for 6 months GOSE means better outcome, while higher OR for survival at discharge means higher chance of survival.

|  | 6 months GOSE  OR (95% CI) | Survival at discharge OR (95% CI) |
| --- | --- | --- |
| Unadjusted | 1.17 (0.86-1.58) | 1.00 (0.63-1.60) |
| Multivariable adjustment* | 1.45 (0.98-2.13) | 1.02 (0.49-2.13) |
| **Subgroup: patients with mass lesion/ASDH** |  |  |
| Unadjusted | 1.64 (1.10-2.44) | 0.87 (0.47-1.61) |
| Multivariable adjustment** | 1.56 (0.99-2.44) | 0.92 (0.31-2.03) |
| **Subgroup: patients with emergency intracranial surgical intervention** |  |  |
| Unadjusted | 1.51 (0.85-2.69) | 0.62 (0.61-0.63) |
| Multivariable adjustment** | 1.62 (0.83-2.73) | 0.67 (0.20-2.25) |

*adjusted for: age, GCS motor score, pupil inequality, hypoxia, hypotension, ISS, CT lesions: tSAH, epidural hematoma, mass lesion, acute subdural hematoma, and a random intercept for center.

** adjusted for: age, GCS motor score, pupil inequality, hypoxia, hypotension, ISS and a random intercept for center.
