# Supplementary figures and images for: Primary versus early secondary referral to a specialized neurotrauma center in patients with moderate/severe traumatic brain injury: a CENTER TBI study
Source: Scand J Trauma Resusc Emerg Med. 2021 Aug 4;29:113. doi: 10.1186/s13049-021-00930-1 (PMC8340517; doi:10.1186/s13049-021-00930-1)

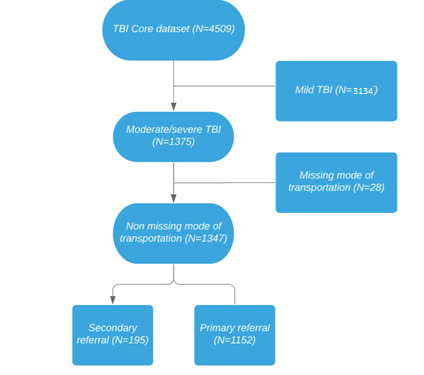

Supplement: Supplementary file 2 — Additional file 2. Figure S1. [file 13049_2021_930_MOESM2_ESM.tif]
